# Resveratrol protects the integrity of alveolar epithelial barrier via SIRT1/PTEN/p‐Akt pathway in methamphetamine‐induced chronic lung injury

**DOI:** 10.1111/cpr.12773

**Published:** 2020-02-05

**Authors:** Xin Wang, Ming Liu, Mei‐Jia Zhu, Lin Shi, Lian Liu, Yuan‐Ling Zhao, Lin Cheng, Ying‐Jian Gu, Ming‐Yuan Zhou, Lei Chen, Ashok Kumar, Yun Wang

**Affiliations:** ^1^ Department of Clinical Pharmacology School of Pharmacy China Medical University Shenyang China; ^2^ Department of Drug Control Criminal Investigation Police University of China Shenyang China; ^3^ Department of Cellular and Molecular Biology University of Texas Health Science Center at Tyler Tyler TX USA

**Keywords:** alveolar epithelial cells, apoptosis, methamphetamine, PTEN, resveratrol, SIRT1

## Abstract

**Objectives:**

SIRT1 is an antioxidative factor, but its mechanism in methamphetamine (MA)‐induced lung injury remains unclear. The purpose of this study is to determine whether MA can disrupt the integrity of alveolar epithelial barrier, whether SIRT1 is involved in MA‐induced chronic lung injury and whether Resveratrol (Res) can protect the integrity of alveolar epithelial cells by regulating ROS to activate SIRT1**/**PTEN/p‐Akt pathway.

**Materials and methods:**

The rats were randomly divided into control group and MA group. Extracted lungs were detected by Western blot, HE staining and immunohistochemistry. The alveolar epithelial cells were treated with MA or/and Res, following by Western blot, LDH leakage assay and flow cytometry. MOE is used for bio‐informatics prediction.

**Results:**

Chronic exposure to MA can cause slower growth ratio of weight, increased RVI and induced lung injury including the reduced number of alveolar sacs and the thickened alveolar walls. MA‐induced apoptosis was associated with SIRT1‐related oxidative stress. Res suppressed ROS levels, activated SIRT1, negatively regulated PTEN, phosphorylated Akt, reduced LDH leakage, increased the expression of ZO‐1 and E‐cadherin and inhibited the apoptosis of alveolar epithelial cells to attenuate MA‐induced higher permeability of alveolar epithelium.

**Conclusions:**

MA disrupted the integrity of alveolar epithelial barrier. Res inhibited oxidative stress and reversed MA‐induced higher permeability and apoptosis of alveolar epithelium by the activation of SIRT1/PTEN/p‐Akt pathway.

## 1 INTRODUCTION

Methamphetamine (MA) is a greatly addictive drug.^1^ MA is abused by snorting, smoking and injection. It is well‐absorbed in pulmonary alveoli.^2^ Alveolar epithelium plays an essential role in maintaining the pulmonary homoeostasis.^3^ Higher uptake rate of MA in lungs suggests that lung is a primary target for MA‐related injury.^4^ It was reported that MA could increase the ROS levels in rat lungs.^5^, ^6^ Excessive accumulation of ROS induces alveolar epithelial apoptosis and even pulmonary toxicity.^5^ Therefore, disruption of alveolar epithelial integrity is the key to chronic lung injury induced by MA.

Sirtuin1 (SIRT1) is an NAD^+^‐dependent deacetylase, which regulates some cellular processes, such as apoptosis, mitochondrial biogenesis, lifespan extension, cell growth and inflammation.^7^, ^8^, ^9^, ^10^, ^11^ SIRT1 can deacetylate histones, non‐histones and some other crucial transcription factors to modulate the ROS production.^10^ Phosphatase and tensin homolog gene (PTEN) is a cancer suppressor with bispecific phosphatase activity and is a multifunctional molecule expressed in various cells.^12^ PTEN function can be negatively regulated by ROS.^13^ PTEN degraded PIP3 and resulted in a decrease in Akt phosphorylation at Ser 473. PTEN is a suppressor of Akt‐mediated signalling in the pulmonary epithelium.^14^ Nonphosphorylated Akt is lack of biological activity. Once it is phosphorylated, p‐Akt is able to regulate cell growth, apoptosis, adhesion, migration, infiltration and metabolism.^15^ Epithelial cells establish closed contacts with their neighbours through intercellular junction complexes (ie tight junctions (TJs) and adherent junctions (AJs)). The TJs of alveolar epithelial cells create a firm intercellular sealing, control the transportation of water and molecules between adjacent cells, and are obligatory to the maintenance of the integrity of the alveolar barrier.^16^ ZO‐1 is one of the important components of TJs. The reduction in ZO‐1 expression has been proven to play a key role in the injury and increased permeability of a variety of epithelium.^17^ Cadherins are the AJs mediating cell–cell adhesion. Epithelial cadherin (E‐cadherin) mediates the adhesion between the adjacent cells.^18^ Disruption and reconstructive failure of alveolar epithelial cells barrier can lead to the catastrophic consequences, such as alveolar congestion and devastating fibrosis. Recent evidences have revealed that the loss of alveolar epithelial integrity is furtherly developed into alveolar mesenchymal transformation.

Resveratrol (3,4',5‐trihydroxystilbene; Res) is a natural polyphenol in grapes, berries, peanuts and other plants.^19^ The functions of Res were reported in cancer, cardiovascular diseases, ischaemic injury and acute poisoning.^20^, ^21^, ^22^ The biological effects of Res may be explained by its antioxidant properties and the activation of SIRT1. SIRT1 is postulated to be a key in the pathophysiology of Res.^19^, ^20^ Based on the above, the current study is aimed to investigated whether MA can disrupt the integrity of alveolar epithelial barrier, whether SIRT1 is involved in MA‐induced chronic lung injury and whether Res can inhibit oxidative stress and protect the integrity of alveolar epithelial cells by activating SIRT1**/**PTEN/p‐Akt pathway.

## 2 MATERIALS AND METHODS

### 2.1 Establishment of animal models

Twenty male Wistar rats (200 ± 10 g) were purchased from Animal Resource Center, China Medical University (Certificate number: Liaoning SCXK 2015‐0001) and were randomly divided into control group and MA‐treated group. MA was from Criminal Investigation Police University of China. At the first week, the rats in the MA group were intraperitoneally injected with 10 mg/kg MA twice/day. Next, the dosage was increased by 1 mg/kg per week, until the daily dosage was increased to 15 mg/kg at the 6th week. The rats in the control group were injected with an equal volume of 0.9% physiological saline solution.^23^ All rats were raised in a room with controlled temperature (18‐22°C) and humidity (50%‐70%) and were fed with solid food and water in an alternating 12 hours light and 12 hours dark cycle. All experimental procedures involving animals were followed by the guidelines of the Guide for the Care and Use of Laboratory Animals of the National Institutes of Health (NIH) with the approval of the Institutional Animal Care and Use Committee of China Medical University (IACUC Issue No. CMU2019215).

### 2.2 Cell culture and treatment

The alveolar epithelial cells A549 (Dingguo Changsheng) were cultured in RPMI 1640 medium (HyClone) supplemented with 10% foetal bovine serum (Clark) and 1% penicillin/streptomycin in 25 cm^2^ culture flask at 37°C in 5% CO_2_. A549 cells were treated by MA with the dosage of 0.1, 0.5, 1 and 5 mmol/L for 6, 12 and 24 hours.^24^ A549 cells were preincubated with N‐acetylcysteine (NAC; N800425, Macklin) or Resveratrol (Res; R817263, Macklin) dissolved in dimethyl sulfoxide (DMSO; D5879, Sigma) for 1 hour before 5 mmol/L MA stimulation. The concentration of DMSO in the medium never exceeded 0.1% to avoid its toxicity towards the A549 cells. To evaluate the effects of NAC, the cells were divided into control group, 5 mmol/L MA group, 5 mmol/L NAC group, and 5 mmol/L MA plus 5 mmol/L NAC group.^25^ To evaluate the effects of Res, the cells were divided into control group, 5 mmol/L MA group, 20 μmol/L Res group and three MA plus Res (1, 5, 20 μmol/L) groups.^26^

### 2.3 HE staining

The inferior lobes of the right lung were fixed with 4% paraformaldehyde buffer overnight, dehydrated, paraffin embedded, sliced into 4 µm sections and stained with haematoxylin and eosin (Hongqiao). The slides were observed under a light microscopy (Olympus BX 51). Lung injury was calculated by the thickness of alveolar septum and the number of alveolar sacs (three visual fields selected randomly were analysed in each section; magnification, ×200 and ×400).^24^

### 2.4 Immunohistochemical assay

The sections were antigen‐retrieved. The slices were, respectively, incubated with the primary antibody of anti‐SIRT1, anti‐E‐cadherin or anti‐ZO‐1 at 4°C overnight. The slides were incubated with goat anti‐rabbit biotinylated secondary antibody (MXB, Fuzhou) for 10 minutes at room temperature and then reacted with streptavidin–peroxidase (MXB, Fuzhou) conjugate for 10 minutes at room temperature. Subsequently, the slices were treated with diaminobenzidine (DAB; Zhongshan Jinqiao) and counterstained with haematoxylin. The slices were dehydrated, mounted and observed under a light microscope.

### 2.5 Western blotting analysis

The lungs or alveolar epithelial cells from different groups were homogenized with ice‐cold RIPA and centrifuged at 12 000 *g* for 15 minutes. Protein concentrations were determined using a BCA Kit (Beyotime). Protein samples were loaded by a SDS–polyacrylamide gel electrophoresis. After blocked with 5% fat‐free milk for 2 hours, the PVDF membranes (GE) were incubated with primary antibodies, respectively, at 4°C overnight (Table 1). Horseradish peroxidase (HRP)‐conjugated goat anti‐rabbit secondary antibodies (Proteintech) were incubated for 2 hours at room temperature. Immunoreactive bands were visualized by DNR Bio‐Imaging systems, and densitometric analysis was determined by imagej software.

**Table 1:** Primary antibodies for Western blot in this study

Primary antibodies	Dilution	Company	Catalogue
SIRT1	1:1000	ABclonal	A11267
PTEN	1:200	BOSTER	BM4114
Akt	1:1000	Proteintech	10176‐2‐AP
p‐Akt‐S473	1:2000	Proteintech	66444‐1‐lg
ZO‐1	1:1000	Proteintech	21773‐1‐AP
E‐cadherin	1:1000	Proteintech	20874‐1‐AP
Bax	1:1000	Proteintech	50599‐2‐lg
Bcl‐2	1:1000	Proteintech	12789‐1‐AP
Caspase 3	1:1000	ABclonal	A11953
Cleaved‐caspase 3	1:1000	ABclonal	A11953
SOD2	1:1000	Proteintech	24127‐1‐AP
GCS	1:1000	Proteintech	12601‐1‐AP
β‐actin	1:2000	Proteintech	66009‐1‐lg

### 2.6 Bio‐informatics prediction

The structures of SIRT1 and PTEN are from RCSB PDB data bank. moe software (CCG) is used to dock model structure of SIRT1 with PTEN. The binding sites between them are analysed by moe software.

### 2.7 Determination of ROS production and cell apoptosis by flow cytometry

ROS levels were quantified using ROS fluorescent probe‐dihydroethidium (DHE; Beyotime) to determine the oxidative stress towards the A549 cells in response to MA stimulation. A549 cells (1 × 10^5^ cells/well) were collected and seeded into 6‐well plates overnight. The cell mono‐layers were washed twice with warmed PBS. The culture medium was replaced with containing 10 μmol/L of DCFH‐DA diluted in RPMI 1640 medium and then incubated in the CO_2_ incubator for 30 minutes at 37°C in humid and dark conditions. A549 cells were harvested and suspended in PBS. Relative fluorescence intensity was analysed by flow cytometry (Becton Dickinson).

Apoptosis was determined by staining cells with FITC‐Annexin V/PI (Vazyme). Cells (1 × 10^6^ cells/well) were collected after the treatments, washed twice with cold PBS and resuspended with 100 μL binding buffer with FITC‐Annexin V (5 μL) and PI staining solution (5 μL). The cells were darkly incubated for 10 minutes at room temperature. The percentage of apoptotic cells was measured by flow cytometry.

### 2.8 LDH assay for the damage of alveolar epithelium

LDH leakage from A549 cells into the culture medium was assessed using an LDH Cytotoxicity Assay Kit (Beyotime). The cells (1 × 10^4^ cells/well) were cultured for 24 hours. Then, the cells from different groups were treated with PBS, 5 mmol/L MA, Res (1, 5, 20 μmol/L) and 20 μmol/L Res + 5 mmol/L MA for another 24 hours at 37°C in a humid incubator, respectively. Subsequent process was according to the supplier's instructions. Finally, the LDH activity was determined using a microplate reader at 490 nm. The LDH leakage rate was calculated by comparing with the LDH activity control group.

### 2.9 Statistical analysis

All the data were expressed as the mean ± standard deviation (SD). Statistical analysis was performed with IBM spss Statistics 22.0 and graphpad prism 6.0 (GraphPad). Differences between two groups were assessed by Student's *t* test, and differences in multiple groups were assessed by one‐way ANOVA. The values of *P* < .05 and *P* < .01 were considered to indicate a statistically significant difference.

## 3 RESULTS

### 3.1 Pulmonary injury induced by chronic exposure to MA

**Figure 1:**
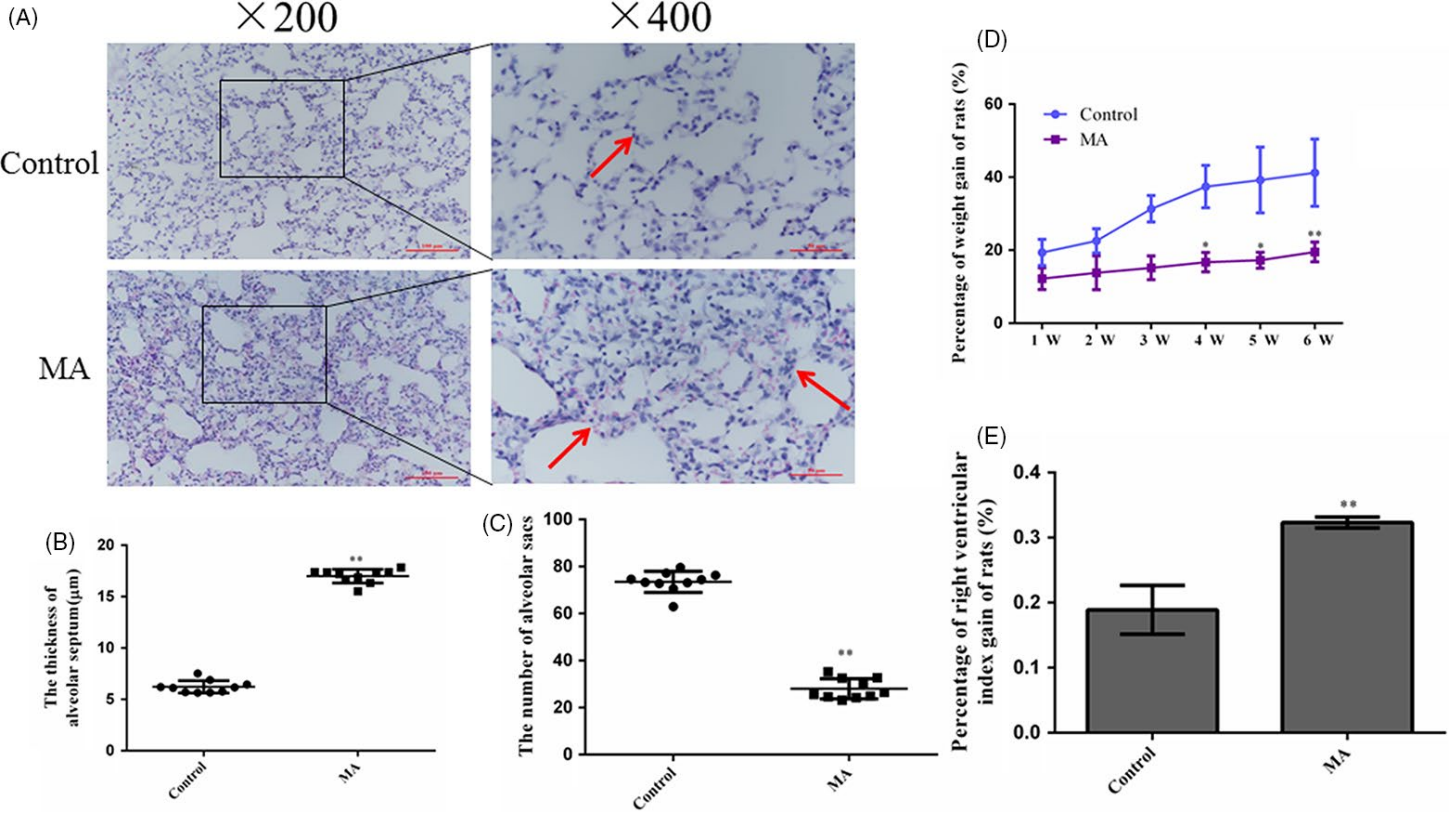
Effects of chronic exposure to MA on pulmonary injury. A, MA induced lung injury by H&E staining (Olympus BX 51, ×200 and ×400). In the MA group, inflammatory cells were infiltrated, lung parenchyma was more compact, alveolar septum was thickened and the number of alveolar sacs was reduced. B, The thickness of alveolar septum. C, The number of alveolar sacs. D, Percentage of weight gain of rats in different groups. E, Percentage of right heart index gain of rats in different groups. The quantification of Figure 1B and Figure 1C was analysed in three visual fields randomly selected in each section, respectively. Data are presented as the mean ± standard deviation (n = 6), ^*^ *P* < .05, ^**^ *P* < .01 vs control group; MA, methamphetamine group

HE staining was used to show pathological changes in rat lungs in different groups. In the control group, the alveolar structure was intact and clear, and there was no inflammatory cell infiltration, bleeding or thickening of the alveolar walls (Figure 1A). But in the MA group, rat lungs showed the marked infiltration of inflammatory cells into the alveolar cavity, more compact parenchyma, reduction in the number of alveolar sacs and the thickened alveolar walls (Figure 1B,C). The percentage of weight gain in the MA group was significantly lower than that in the control group from the 4th week to 6th week (Figure 1D). Right ventricular index (RVI) 0.18 ± 0.037 from the control group was obviously increased to 0.32 ± 0.008 in the MA group (^**^ *P* < .01, Figure 1E).

### 3.2 MA disrupted the integrity of alveolar epithelial barrier

**Figure 2:**
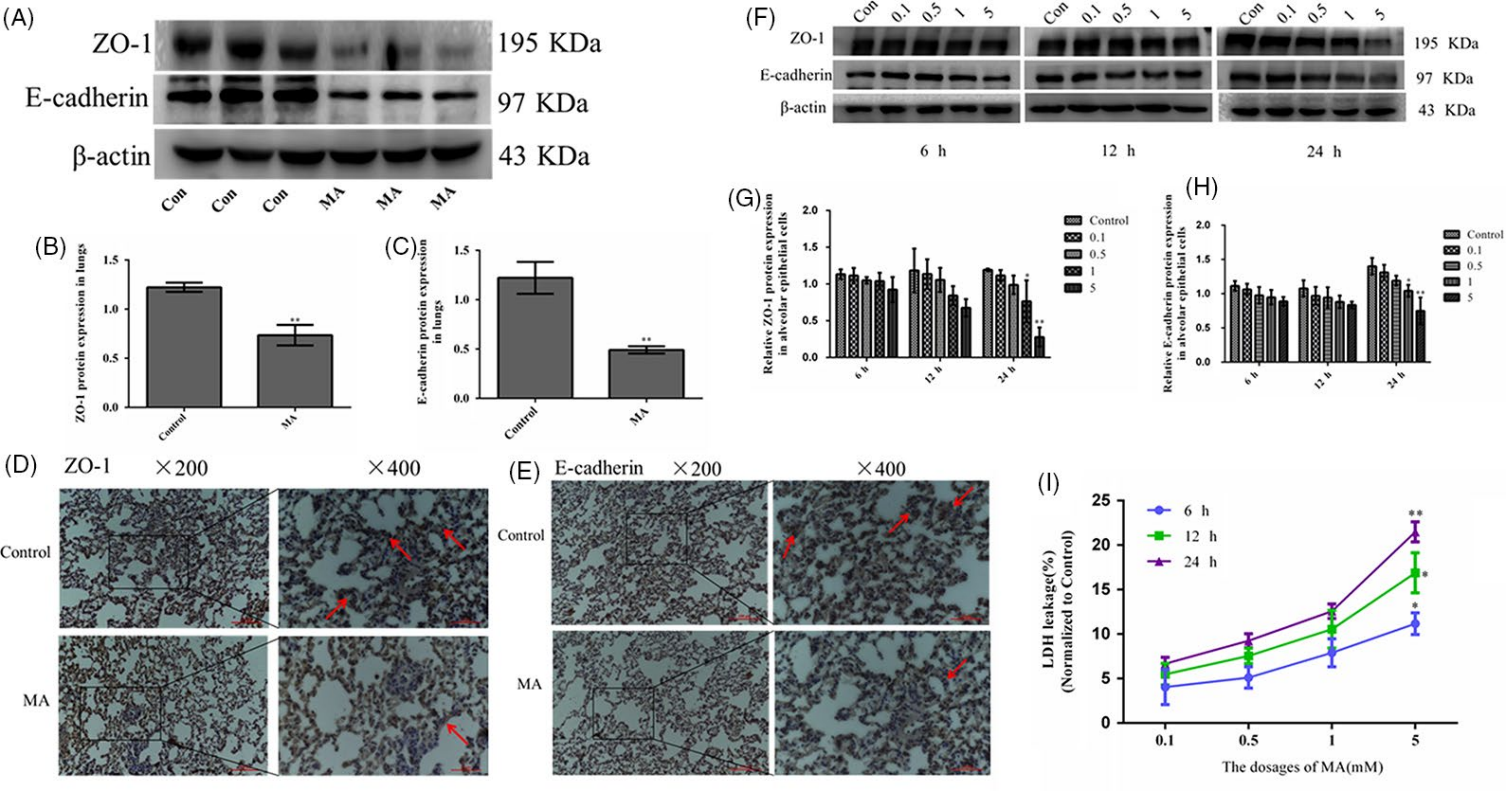
MA disrupted the integrity of alveolar epithelial barrier. A‐C, The protein expression levels of ZO‐1 and E‐cadherin were determined by Western blot. D, Expression of ZO‐1 in lungs in different groups by immunohistochemical staining. E, Expression of E‐cadherin in lungs in different groups by immunohistochemical staining. F‐H, ZO‐1 and E‐cadherin expressed in alveolar epithelial cells. I, The effects of MA on leakage of LDH from alveolar epithelial cells (representing membrane damage) at 6, 12 and 24 h. Data are presented as the mean ± standard deviation. ^*^ *P* < .05, ^**^ *P* < .01, vs control group. 0.1, 0.1 mmol/L, 0.5, 0.5 mmol/L; 1, 1 mmol/L; 5, 5 mmol/L; MA, methamphetamine

To determine whether MA can increase the permeability of alveolar epithelium, it is necessary that TJ protein ZO‐1 and AJ protein E‐cadherin should be detected. Western blot analysis showed that E‐cadherin and ZO‐1 in lungs were dramatically decreased in the MA group, compared with the control group (Figure 2A‐C). In Figure 2D, ZO‐1 was expressed higher and localized at tight junctions in the control group, but in the MA group, ZO‐1 expression was obviously diminished. And a significant downregulation of E‐cadherin level was also observed in the MA group (Figure 2E). After A549 cells were treated with MA (0.1, 0.5, 1, 5 mmol/L) for 6, 12 and 24 hours, it was found that MA reduced the levels of ZO‐1 and E‐cadherin at time‐ and dose‐dependent manners (Figure 2F). Especially, there were marked reductions in ZO‐1 and E‐cadherin with 5 mmol/L MA at 24 hours (Figure 2G,H). Additionally, 5 mmol/L MA caused a remarkable increase in LDH leakage in alveolar epithelial cells at 24 hours, compared with the control group (Figure 2I). These results indicated that chronic exposure of MA can increase the permeability of cell epithelium to disrupt the barrier function of alveolar epithelium.

### 3.3 MA‐induced oxidative stress and apoptosis of alveolar epithelial cells

**Figure 3:**
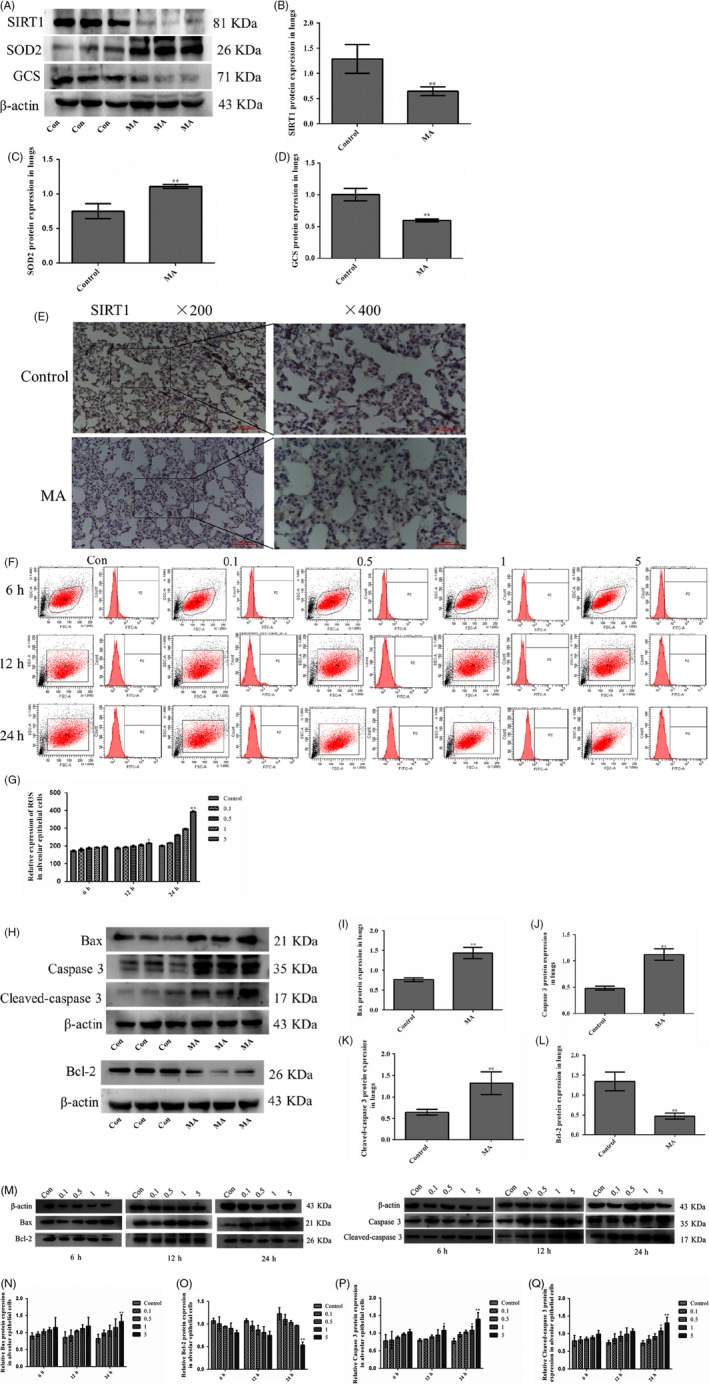
MA‐induced oxidative stress and apoptosis of alveolar epithelial cells. A‐D, Effect of MA on the expression of SIRT1, SOD2 and GCS in alveolar epithelial cells. E, Expression of SIRT1 in lungs by immunohistochemical staining. (F‐G) ROS levels in the alveolar epithelial cells by flow cytometry. H‐L, The expression of Bax, caspase 3, cleaved‐caspase 3 and Bcl‐2 in lungs. M‐Q, The expression of Bax, caspase 3, cleaved‐caspase 3 and Bcl‐2 in alveolar epithelial cells. Data are presented as the mean ± standard deviation. ^*^ *P* < .05, ^**^ *P* < .01, vs control group. 0.1, 0.1 mmol/L, 0.5, 0.5 mmol/L; 1, 1 mmol/L; 5, 5 mmol/L; MA, methamphetamine

SIRT1 can modulate the ROS levels to protect against oxidative stress‐mediated injury. Western blot analysis showed that SIRT1 expression in the control group was higher than that in the MA group (Figure 3A,B), which was consistent with the tendency of SIRT1 expression by IHC (Figure 3E). Oxidative enzyme superoxide dismutase (SOD) reflects the degree of oxidative stress injury. In our study, it was found that SOD2 in lungs was highly expressed in the MA groups (Figure 3C). Antioxidative enzyme glutamylcysteine synthetase (GCS) can scavenge oxygen free radicals, so its expression was reduced by MA (Figure 3D). Excessive production of ROS is the main cause of oxidative stress. Flow cytometry was used to analyse the levels of ROS in living cells by measuring 10 000 cells (Figure 3F). In flow cytometry analysis, MA elevated the production of intracellular ROS, and even at 24 hours, the ROS level was increased approximately twice as much as that in the control group (P1 from 205 in the control group to 389 in 5 mmol/L MA; Figure 3G). Excessive oxidative stress induces the cell apoptosis. The apoptosis of alveolar epithelial cells is an important pathophysiological process of an increase in the permeability of alveolar epithelial barrier. In rat lungs, apoptotic proteins caspase 3, cleaved‐caspase 3 and the proapoptotic protein Bax were significantly upregulated by MA, but antiapoptotic protein Bcl‐2 was dramatically downregulated by MA (Figure 3H‐L). Meanwhile, in alveolar epithelial cells, caspase 3, cleaved‐caspase 3 and Bax were significantly increased, but Bcl‐2 was markedly reduced by 5 mmol/L MA at 24 hours (Figure 3M‐Q).

**Figure 4:**
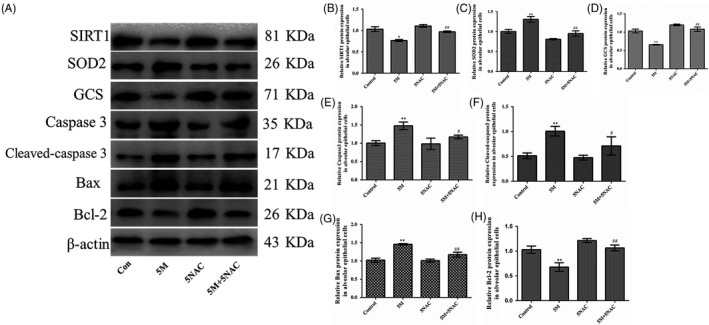
The effects of ROS inhibitor on SIRT1 and apoptosis of alveolar epithelial cells by MA. A, Effects of NAC on the protein expression of SIRT1, oxidative stress and apoptotic‐related factors by Western blot. B‐H, The protein expression of SIRT1, SOD2, GCS, caspase 3, cleaved‐caspase 3, Bax and Bcl‐2 in alveolar epithelial cells. Data are presented as the mean ± standard deviation. ^*^ *P* < .05, ^**^ *P* < .01 vs control group, ^#^ *P* < .05, ^##^ *P* < .01 vs 5M group. 5M, 5 mmol/L methamphetamine; 5NAC, 5 mmol/L N‐acetylcysteine; 5M + 5NAC, 5 mmol/L methamphetamine and 5 mmol/L N‐acetylcysteine

To determine the correlation between ROS, SIRT1 and apoptosis, alveolar epithelial cells were treated with the ROS scavenger NAC with the dose of 5 mmol/L (5NAC) or/and MA with the dose of 5 mmol/L (5M) for 24 hours (Figure 4A). It was found that the expression of SIRT1 was significantly reversed from MA by NAC (Figure 4B). NAC effectively blocked MA‐induced oxidative stress (Figure 4C,D). Meanwhile, the levels of caspase 3, cleaved‐caspase 3 and Bax were markedly downregulated and Bcl‐2 was obviously upregulated in the 5M + 5NAC group, compared with the 5M group (Figure 4E‐H). These results were confirmed that SIRT1 expression and apoptosis of alveolar epithelial cells were modulated by MA‐induced oxidative stress.

### 3.4 Res reversed MA‐induced ROS production and apoptosis

**Figure 5:**
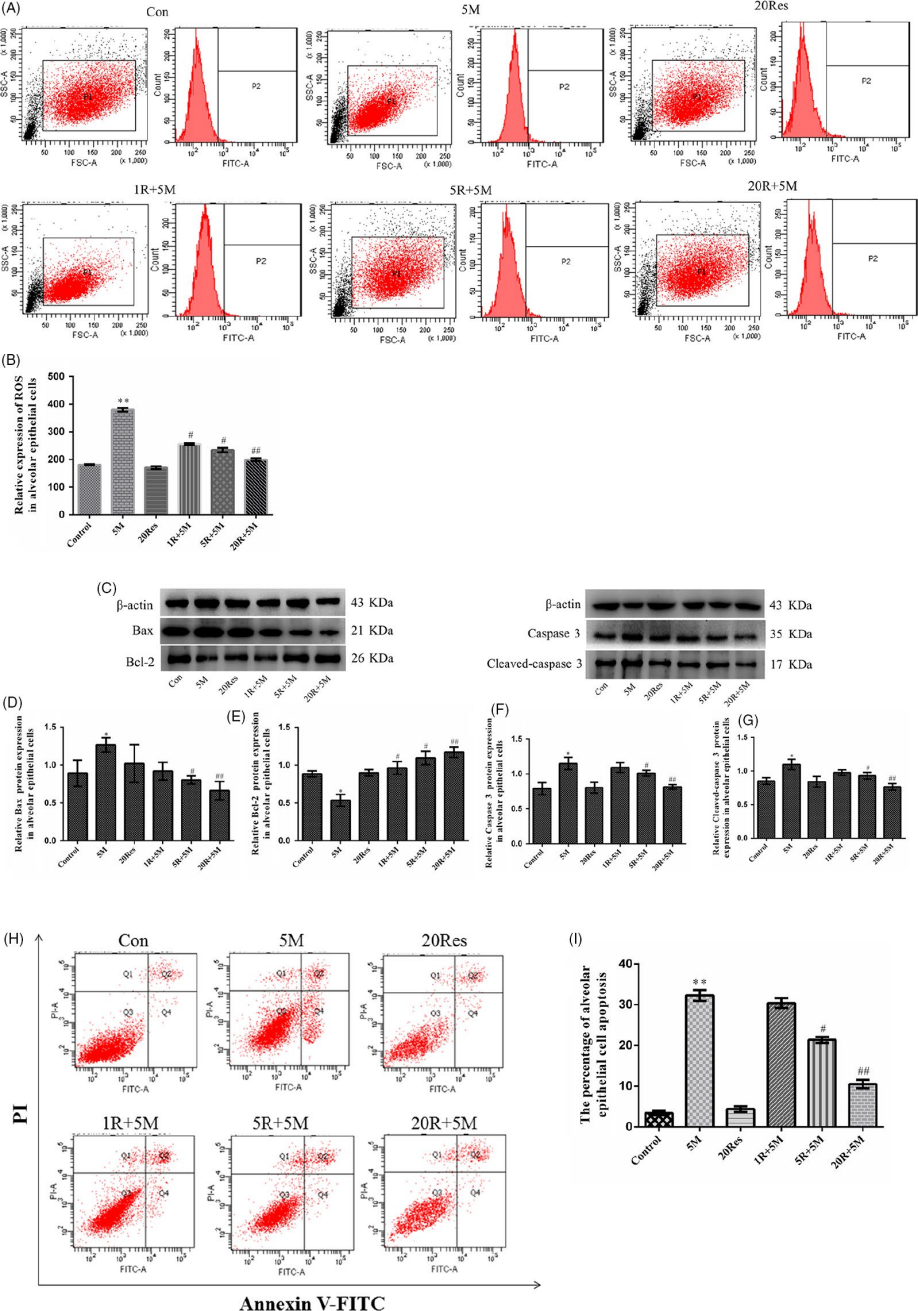
Res reversed MA‐induced ROS production and apoptosis. A‐B, Effects of Res on ROS production by flow cytometry. C‐G, Effects of Res on the expression of Bax, Bcl‐2, caspase 3 and cleaved‐caspase 3 in alveolar epithelial cells. A549 cells were pre‐treated with Res (1, 5, 20 μmol/L) for 1 h and then incubated with 5 mmol/L MA for 24 h. H‐I, Effects of Res on the apoptosis of the alveolar epithelial cells. The cells were stained with FITC‐Annexin V/PI and then analysed by flow cytometry. Data are presented as the mean ± standard deviation. ^*^ *P* < .05 ^**^ *P* < .01 vs control group, ^#^ *P* < .05, ^##^ *P* < .01 vs 5M group. 5M, 5 mmol/L methamphetamine; 20Res, 20 μmol/L Resveratrol; 1R + 5M, 1 μmol/L Resveratrol and 5 mmol/L methamphetamine; 5R + 5M, 5 μmol/L Resveratrol and 5 mmol/L methamphetamine; 20R + 5M, 20 μmol/L Resveratrol and 5 mmol/L methamphetamine

In order to furtherly determine MA‐induced apoptosis by SIRT1‐related oxidative stress, in the present study, the alveolar epithelial cells were pre‐treated with Res, a SIRT1 activator, previous to MA and were divided into six groups: control, 5 mmol/L MA (5M), 20 μmol/L Res (20Res), 5M plus Res (1, 5 and 20 μmol/L) groups. Flow cytometry analysis showed that 5 mmol/L MA increased the production of ROS compared with the control group (P1 increased from 179 to 312). Res alone reduced ROS level without statistical significance compared with the control group. But the production of ROS was partially reversed by 20 μmol/L Res from MA (P1: 312 in the 5M group vs 200 in the 20Res + 5M group; Figure 5A,B). It was also found that Res significantly decreased Bcl‐2, caspase 3 and cleaved‐caspase 3, and increased Bax, compared with the 5M group (Figure 5C‐G). FITC‐Annexin V/PI results showed that MA markedly induced the apoptosis of alveolar epithelial cells; however, 20 μmol/L Res significantly reversed the percentage of the cellular apoptosis from MA (32.3% in the 5M group vs 10.5% in the 20Res + 5M group; Figure 5H,I). These results confirmed that Res markedly alleviated oxidative stress and reversed MA‐induced apoptosis of alveolar epithelial cells.

### 3.5 Bio‐informatics prediction of interaction between SIRT1 and PTEN

**Figure 6:**
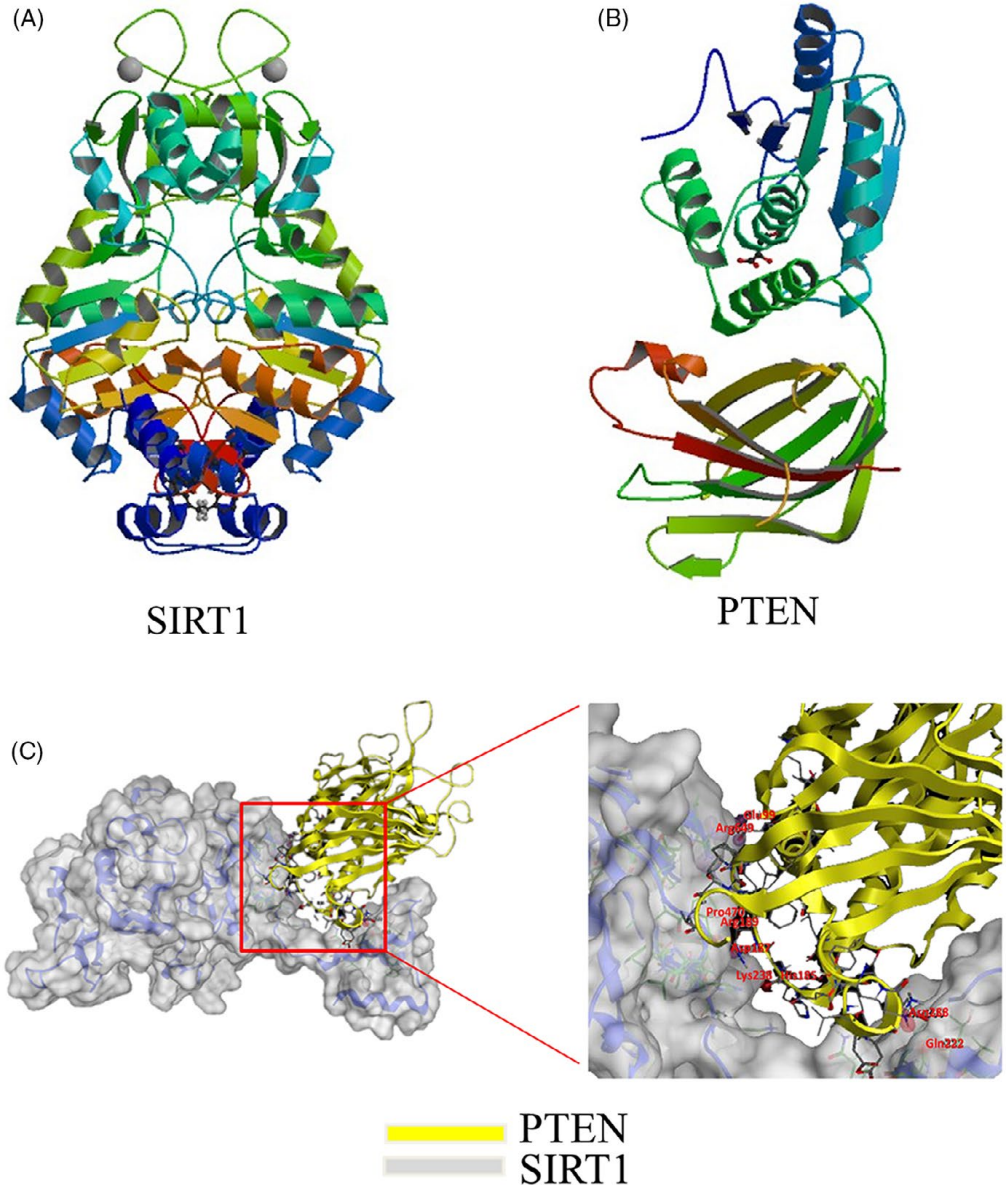
Bio‐informatics prediction of interaction between SIRT1 and PTEN. A, Molecular model of SIRT1, obtained from the PDB website. B, Molecular model of PTEN, obtained from the PDB website. C, Molecular docking of SIRT1 with PTEN by moe software. moe analysis showed the binding sites of docking structure of SIRT1 to PTEN

SIRT1 can regulate the expression of PTEN,^27^ but it is unknown if SIRT1 can interact with PTEN. To further explore this possibility, we modelled the structures of SIRT1 and PTEN (Figure 6A,B) from RCSB PDB data bank, and further docked SIRT1 with PTEN by moe software (Figure 6C). In moe analysis, PTEN docked in the C‐terminal domain of SIRT1. The binding sites of hydrogen bonds between SIRT1 and PTEN were shown in Table 2. It was illustrated that SIRT1 can interact with PTEN and directly regulate the expression of PTEN.

**Table 2:** The binding sites of hydrogen bonds between SIRT1 and PTEN

SIRT1	PTEN
Arg649	Glu99
Gln222	Asn288
Pro470	Arg189
His185	Asp187

### 3.6 Res alleviated the disruption of alveolar epithelial integrity through SIRT1/PTEN/p‐Akt pathway

**Figure 7:**
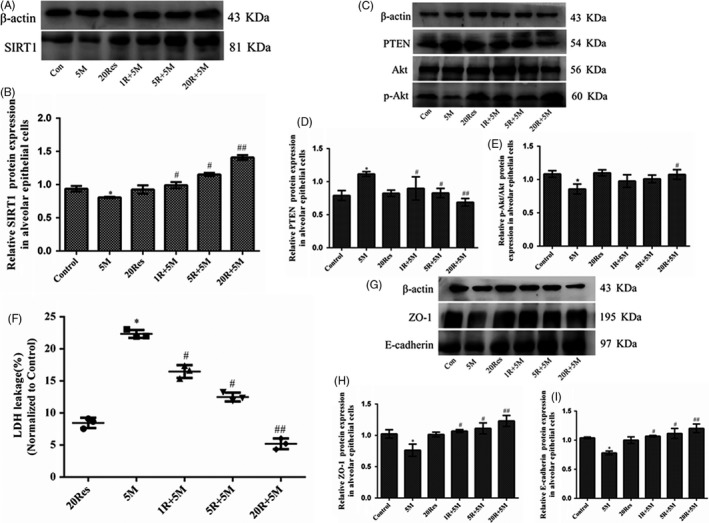
Res alleviated the disruption of the alveolar epithelial integrity through SIRT1/PTEN/p‐Akt pathway. A‐B, Effects of Res on SIRT1 expression in alveolar epithelial cells by Western blot. C‐E, Effects of Res on the protein expression of PTEN, Akt and p‐Akt in alveolar epithelial cells. F, The effects of Res on leakage of LDH from alveolar epithelial cells. A549 cells were pre‐treated with Res (1, 5, 20 μmol/L) for 1 h and then incubated with 5 mmol/L MA for 24 h. G‐I, Effects of Res on the protein expression of ZO‐1 and E‐cadherin in alveolar epithelial cells. Data are presented as the mean ± standard deviation.^*^ *P* < .05 vs control group, ^#^ *P* < .05, ^##^ *P* < .01 vs 5M group. 5M, 5 mmol/L methamphetamine; 20 Res, 20 μmol/L Resveratrol; 1R + 5M, 1 μmol/L Resveratrol and 5 mmol/L methamphetamine; 5R + 5M, 5 μmol/L Resveratrol and 5 mmol/L methamphetamine; 20R + 5M, 20 μmol/L Resveratrol and 5 mmol/L methamphetamine

According to the possible interaction between SIRT1 and PTEN, Res, a SIRT1 activator, was used to detect the effect of SIRT1 on PTEN. It was found that SIRT1 was dose dependently increased by pre‐treated Res and that the increase in SIRT1 expression was particularly significant by 20 μmol/L Res (Figure 7A,B). In addition, 20 µmol/L Res obviously reduced the expression of PTEN and accelerated the phosphorylation of Akt (Figure 7C‐E). LDH leakage was used to analyse the permeability of cell membranes. The results from this study showed that MA caused a remarkable increase in LDH leakage of alveolar epithelial cells, compared with the control group; however, 20 μmol/L Res markedly reversed the increasing of LDH leakage induced by 5 mmol/L MA (Figure 7F). Additionally, the expression of ZO‐1 and E‐cadherin was obviously increased in the Res + 5M groups from that in the 5M group (Figure 7G‐I). These results were suggested that Res can alleviate MA‐induced disruption of alveolar epithelial integrity through SIRT1/PTEN/p‐Akt pathway.

## 4 DISCUSSION

Chronic exposure to MA can cause slower growth ratio of weight, increased RVI and induced lung injury including more compact lung parenchyma, the reduced number of alveolar sacs and the thickened alveolar walls. Res inhibited oxidative stress by suppressing ROS generation. Res activated SIRT1, negatively regulated PTEN, phosphorylated Akt, reduced LDH leakage, increased the expression of ZO‐1 and E‐cadherin, decreased the levels of caspase 3, cleaved‐caspase 3 and Bax, upregulated Bcl‐2, and reduced the percentage of the apoptosis of alveolar epithelial cells to attenuate MA‐induced higher permeability of alveolar epithelium. These results suggested that MA induced the lack of alveolar epithelial integrity and that Res reversed MA‐induced oxidative stress, higher permeability and apoptosis of alveolar epithelial cells via SIRT1/PTEN/p‐Akt pathway.

The chronic accumulation of MA resulted in the pulmonary complications attributed to its high uptake in lungs.^5^, ^28^ Lung toxicity includes alveolar oedema, exudation, thickening of the alveolar septa and inflammatory cell infiltration.^29^ In our study, it was found of more compact lung parenchyma, reduction in the number of alveolar sacs, the thickened alveolar walls and infiltration of inflammatory cells, which suggested that the damages of alveolar epithelial integrity and an increase in the permeability of alveolar epithelial cells are involved in MA‐induced lung injury.

Pulmonary epithelium as a barrier limits the access of various toxic to the respiratory system to maintain the pulmonary homoeostasis.^30^ Therefore, alveolar epithelial cells are also considered to play an important role in the defence and anti‐infection to reduce the surface tension of the alveoli, promote the gas exchanges and improve the restoration of lungs.^13^ TJs and AJs, such as ZO‐1 and E‐cadherin, are located on the apical surface of the sidewalls of adjacent alveolar epithelial cells.^17^ ZO‐1 and E‐cadherin provide the linkage between the cell membranes, and they are essential for the integrity of alveolar epithelial barrier.^17^ The results from our study were that the epithelial barrier markers ZO‐1 and E‐cadherin were localized at the alveolar walls and that MA dramatically decreased the expressions of E‐cadherin and ZO‐1 in lungs and in alveolar epithelial cells, which reflected that MA induced the lack of the function of alveolar epithelial barrier.

Sirtuins are a unique of histone III deacetylases with seven subtypes SIRT1‐7.^31^ SIRT1 is a NAM adenine dinucleotide (NAD^+^)‐dependent histone deacetylase involved in multiple cellular functions, for example, oxidative stress.^32^, ^33^, ^34^, ^35^ Oxidative stress is mediated by ROS.^3^ Some studies have revealed that there is a crosstalk between SIRT1 and oxidative stress.^36^, ^37^ It was founded that MA inhibited the expression of SIRT1 by inducing oxidative stress with elevated ROS levels, increased SOD and reduced GCS. MA‐induced lung toxicity is linked to oxidative injury by directly activating NAPDH oxidase and generating of ROS that cause cellular damages.^10^, ^38^ Previous studies reported that MA increased ROS production in lungs,^24^ which are consistent with the results of the current study. Our results suggested that long‐term exposure to MA significantly decreased the expression of SIRT1 in response to oxidative stress in vivo and vitro. Apoptosis of alveolar epithelial cells is an important pathogenesis of lung injury.^39^ In alveolar epithelial cells treated with MA, it was found of upregulation of apoptosis factor, caspase 3, cleaved‐caspase 3 and Bax, and downregulation of apoptosis inhibitor Bcl‐2, which was illustrated that long‐term exposure to MA can damage alveolar epithelial barrier by inducing apoptosis. The structural integrity of the alveolar epithelial mono‐layer is regulated by the balance between inward tension forces and outward adhesive tethering forces at cell–cell and cell–matrix contacts.^40^ LDH assay can be used to evaluate the integrity or the permeability of the alveolar epithelium. The result from this study is that 5 mmol/L MA caused a remarkable increase in LDH leakage in alveolar epithelial cells at 24 hours. To determine the correlation between oxidative stress, SIRT1 and apoptosis, alveolar epithelial cells were incubated with 5mM NAC (5NAC) or/and 5 mmol/L MA (5M) for 24 hours. It was found that the expression of SIRT1 and oxidative stress was significantly reversed from MA by NAC. Meanwhile, the expression of caspase 3, cleaved‐caspase 3 and Bax was markedly increased and Bcl‐2 was obviously reduced in the 5M + 5NAC group, compared with the 5M group. These results were confirmed that SIRT1 expression and apoptosis of alveolar epithelial cells were modulated by MA‐induced oxidative stress.

In order to furtherly determine that MA‐induced apoptosis is associated with SIRT1‐related oxidative stress, the alveolar epithelial cells were pre‐treated with Res, a SIRT1 activator, previous to MA. Flow cytometry analysis showed that the production of ROS was partially reversed by 20 μmol/L Res from MA. It was also found that in Res significantly decreased Bcl‐2, caspase 3 and cleaved‐caspase 3, and increased Bax, compared with the group of 5 mmol/L MA. FITC‐Annexin V/PI results showed that MA markedly induced the apoptosis of alveolar epithelial cells, and Res significantly reversed the percentage of the cellular apoptosis from MA. The above results were indicated that MA induced in‐completion of alveolar epithelial barrier with apoptosis by SIRT1‐related oxidative stress.

PTEN can dephosphorylate PIP3 into PIP2 and PIP2 to PIP to switch off the pathway of Akt phosphorylation, and then downregulated the levels of p‐Akt.^41^, ^42^, ^43^ A major mechanism in the cellular oxidative stress is the activation of the PTEN/p‐Akt signalling pathway.^44^ SIRT1 can regulate the expression of PTEN,^27^ but it is unknown if SIRT1 can interact with PTEN. To further explore it, we modelled the structures of SIRT1 and PTEN from RCSB PDB, and further docked SIRT1 with PTEN by MOE software. In MOE analysis, PTEN docked in the C‐terminal domain of SIRT1. The binding sites of hydrogen bonds between SIRT1 and PTEN were shown in Table 2, which indicated that SIRT1 can interact with PTEN and directly regulate the expression of PTEN. In present study, MA reduced SIRT1, but increased PTEN, and prevented the phosphorylation of Akt in vitro and in vivo. The results further indicated that SIRT1 downregulation resulted in the increasing expression of PTEN and decreasing expression of p‐Akt. Res, a SIRT1 activator, upregulated the expression of SIRT1 accompanied by inactivation of PTEN/p‐Akt pathway and reduced production of ROS. These results were clarified that SIRT1/PTEN/p‐Akt pathway plays a key role in regulating oxidative stress. Meanwhile, it was found that Res inhibited apoptosis of alveolar epithelial cells by downregulating Bax, caspase 3, cleaved‐caspase 3 and upregulating Bcl‐2 and that Res retrieved the barrier function of alveolar epithelium by preventing LDH leakage and increasing ZO‐1 and E‐cadherin expression. Therefore, Res exerted the protective effects on the integrity of alveolar epithelial barrier via SIRT1/PTEN/p‐Akt pathway.

In summary, chronic exposure to MA can disrupt the integrity of alveolar epithelial barrier. Resveratrol can inhibit oxidative stress and reverse MA‐induced higher permeability and apoptosis of alveolar epithelium via SIRT1/PTEN/p‐Akt pathway.

## CONFLICT OF INTEREST

The authors declare that there are no competing interests.

## AUTHOR CONTRIBUTION

Xin and Yun designed the study; Ming and Yun contributed the new reagents or analytical tools; Xin, Ming, Mei‐Jia, Lin, Lian, Yuan‐Ling, Ming‐Yuan and Lei collected the data; Xin, Ming, Lin and Ying‐Jian analysed the data; Xin and Yun prepared the manuscript; Ashok and Yun modified the language.

## Data Availability

The data that support the findings of this study are available from the corresponding author upon reasonable request.
